# Latent profile analysis of professional identity and its correlation with humanistic practice competence in undergraduate nursing interns

**DOI:** 10.3389/fmed.2025.1663870

**Published:** 2025-12-19

**Authors:** Shuqin Xu, Yuanyuan Gao, Yixun Zou, Jingying Shen, Aiyong Zhu, Chong Zhao, Qianqian Zhou

**Affiliations:** 1 School of Nursing and Health Management, Shanghai University of Medicine & Health Sciences, Shanghai, China; 2 School of Clinical Medicine, Shanghai University of Medicine & Health Sciences, Shanghai, China; 3 Department of Orthopedic Ward II, Shanghai Ninth People’s Hospital, Shanghai JiaoTong University School of Medicine, Shanghai, China; 4 Department of Physical Education, Shanghai University of Medicine & Health Sciences, Shanghai, China

**Keywords:** clinical education, correlation study, humanistic practice competence, latent profile analysis, professional identity, undergraduate nursing interns

## Abstract

**Introduction:**

Nursing professional identity is a key predictor of clinical performance and career development among nursing interns, with its heterogeneity being particularly pronounced during clinical placements. Current research often overlooks subgroup differences and lacks exploration of the mechanisms linking nursing professional identity to humanistic practice competence, which hampers the development of targeted educational strategies. This study aims to identify and explore the latent categories and characteristics of professional identity among interns, analyze influencing factors across different categories, and provide references for developing targeted nursing education interventions.

**Methods:**

Using convenience sampling, 295 undergraduate nursing interns from Shanghai University of Medicine & Health Sciences were surveyed between December 2023 and April 2025. The investigation utilized a general information questionnaire, the Nurse Professional Identity Scale, the Nurse Humanistic Practice Competence Assessment Scale. Latent profile analysis was employed to identify potential categories of professional identity, and logistic regression analysis was used to determine its influencing factors.

**Results:**

Professional identity was categorized into three latent profiles: low-level group (35.25%), moderate-level group (49.50%), and high-level group (15.25%). Multivariate analysis revealed that current appreciation for the nursing profession, reasons for choosing nursing as major (parental advice), self-management ability, and ethical and legal practice ability were significant predictors of professional identity levels (*p* < 0.05).

**Discussion:**

The professional identity of nursing interns is heterogeneous, with the majority categorized at moderate or low levels. Personal attitudes and core competencies are key influencing factors. Educators should implement stratified interventions based on the three identified profiles: For the low-level group, enhance emotional support and foster professional identity through narrative pedagogy and micro-goal mentoring. For the moderate-level group, boost reflective practice and intrinsic motivation via structured volunteer-clinical linkages. For the high-level group, promote sustained development through research innovation and dual mentorship. Furthermore, integrating self-management and ethical practice training into the curriculum, along with pre-admission career experience programs, is recommended to effectively enhance professional identity and humanistic practice competence.

## 1 Introduction

Nursing professional identity refers to nursing personnel’s cognition and acceptance of their professional roles, values, and missions. It directly influences career stability, work quality, and patient safety (1). The construction of the Nurse Professional Identity Scale was theoretically informed by Self-Determination Theory (SDT) (2) and Identity Status Theory (IST) (3). SDT posits that human behavior is driven by three basic psychological needs: autonomy, competence, and relatedness. The extent to which these needs are satisfied influences the internalization of motivation and an individual’s sense of well-being, thereby shaping their identification with a particular role or identity. SDT explains the “motivational source” of professional identity: positive major choice motivation satisfies nursing students’ need for autonomy (feeling they have chosen and endorse the major) and competence (feeling capable and able to grow). IST focuses on individual differences along two dimensions—exploration and commitment—forming different identity statuses (e.g., foreclosure, diffusion, moratorium, achievement). These statuses are dynamic and influenced by experiences and cognition. IST describes the “formation process” of professional identity: the process of career understanding itself constitutes a form of “exploration,” and positive major choice motivation promotes active exploration, leading to “commitment” based on that exploration. As a result, individuals are more likely to achieve the “identity achievement” status, thereby strengthening their professional identity. The scale’s dimensions collectively capture the motivational and developmental processes of identity formation. Specifically, professional cognitive evaluation, social skills, and social support are central to fulfilling the SDT needs for relatedness and autonomy, as they foster a sense of belonging, volitional control, and intrinsic motivation. These dimensions help students develop a sense of belonging, volitional control, and intrinsic motivation. Meanwhile, professional self-reflection embodies the exploratory process vital to IST, enabling individuals to critically examine their role and progress from uncertainty toward commitment. As for professional frustration coping, it directly contributes to a sense of competence SDT by reinforcing one’s belief in their professional efficacy through effectively managing challenges. Thus, the scale provides a multifaceted measure that is conceptually rooted in these established frameworks of motivation and identity development. Nurses with stronger professional identity demonstrate better clinical decision-making abilities and humanistic care behaviors than do their counterparts (4). As nursing models and public health concepts evolve, humanistic care has gained increasing attention. This development places higher demands on nurses’ humanistic practice competence (5). Humanistic practice competence in this context refers to the ability and skill of nursing professionals to apply humanistic knowledge in clinical work. It is an external manifestation of their inner humanistic concepts, knowledge, skills, and spirit. This competence complements clinical treatment and nursing abilities, working alongside them as an equally vital component (6).

Professional identity and humanistic practice competence are conceptualized as distinct yet synergistic constructs. Drawing on existing literature, professional identity is defined as an internal, psychological state of belonging and value internalization [e.g., (1)], whereas humanistic practice competence constitutes an external, behavioral manifestation of skills required to deliver patient-centered care [e.g., (6)]. According to SDT, strong humanistic practice competency can significantly fulfill nursing students’ need for relatedness (building positive relationships with patients and colleagues) and competence (feeling capable of helping others professionally). When they experience positive feedback and emotional connection in practice as a result of their own abilities, their work motivation becomes more autonomous and internalized, thereby strengthening their professional identity. From the perspective of IST, well-developed humanistic practice competency helps nursing students engage in more effective “professional role exploration” during interactions with patients. This facilitates their transition from the “exploration” stage to the “commitment” stage, leading to “identity achievement” and consolidating the stability of their professional identity. We posit a reciprocal and mutually reinforcing relationship between them. Specifically, professional identity likely acts as an antecedent and motivator for developing humanistic competence, as individuals are driven to act in ways that align with their internalized values (2). Conversely, humanistic competence reinforces professional identity through a sense of efficacy and achievement derived from its successful application—a process aligned with the concept of mastery experiences in self-efficacy theory (7). Therefore, although our current study treats them as correlated dimensions, the described temporal and functional dynamics suggest that their relationship could be effectively modeled in future research as a reciprocal mediation within a broader competence development framework.

However, the internship stage is a critical period for the formation of professional identity among undergraduate nursing students, and fluctuations in their identity levels may affect future career choices (8). As undergraduate nursing students are an important reserve force for China’s nursing cause, paying attention to their career choices is crucial for the development of the industry. Current domestic studies on professional identity among nursing students mostly rely on traditional linear models or group comparisons, ignoring the heterogeneity of individual characteristics and making it difficult to accurately identify the differentiated needs of various groups (9). Latent profile analysis (LPA) identifies latent subgroups through manifest variables. This approach allows for a more detailed analysis of internal distribution characteristics within groups, thereby providing a basis for personalized interventions (9, 10). Against this background, LPA is particularly suitable for examining the heterogeneity of professional identity. First, LPA is a person-centered analytical method that identifies latent subgroups (profiles) based on the combination of manifest variables (e.g., scores on multiple dimensions of professional identity), rather than focusing on variable-centered relationships. This aligns with the multi-dimensional and heterogeneous nature of professional identity, as it reveals how different dimensions interact to form distinct identity patterns (e.g., high scores on all dimensions vs. low professional cognition but moderate frustration coping). Second, LPA avoids the limitations of traditional classification methods (e.g., arbitrary cut-off points) by using statistical criteria to determine the optimal number of subgroups, ensuring the objectivity and validity of classification (10). Third, LPA can explicitly describe the characteristics of each subgroup (e.g., proportion, dimension scores) and analyze the influencing factors of subgroup membership, providing a direct basis for personalized and stratified educational strategies. This study employs LPA to identify latent categories and characteristics of professional identity among undergraduate nursing interns and examines its correlation with humanistic care competence. The findings aim to offer a theoretical basis for nursing educators to develop stratified training strategies for enhancing students’ professional identity.

**Figure 1:**
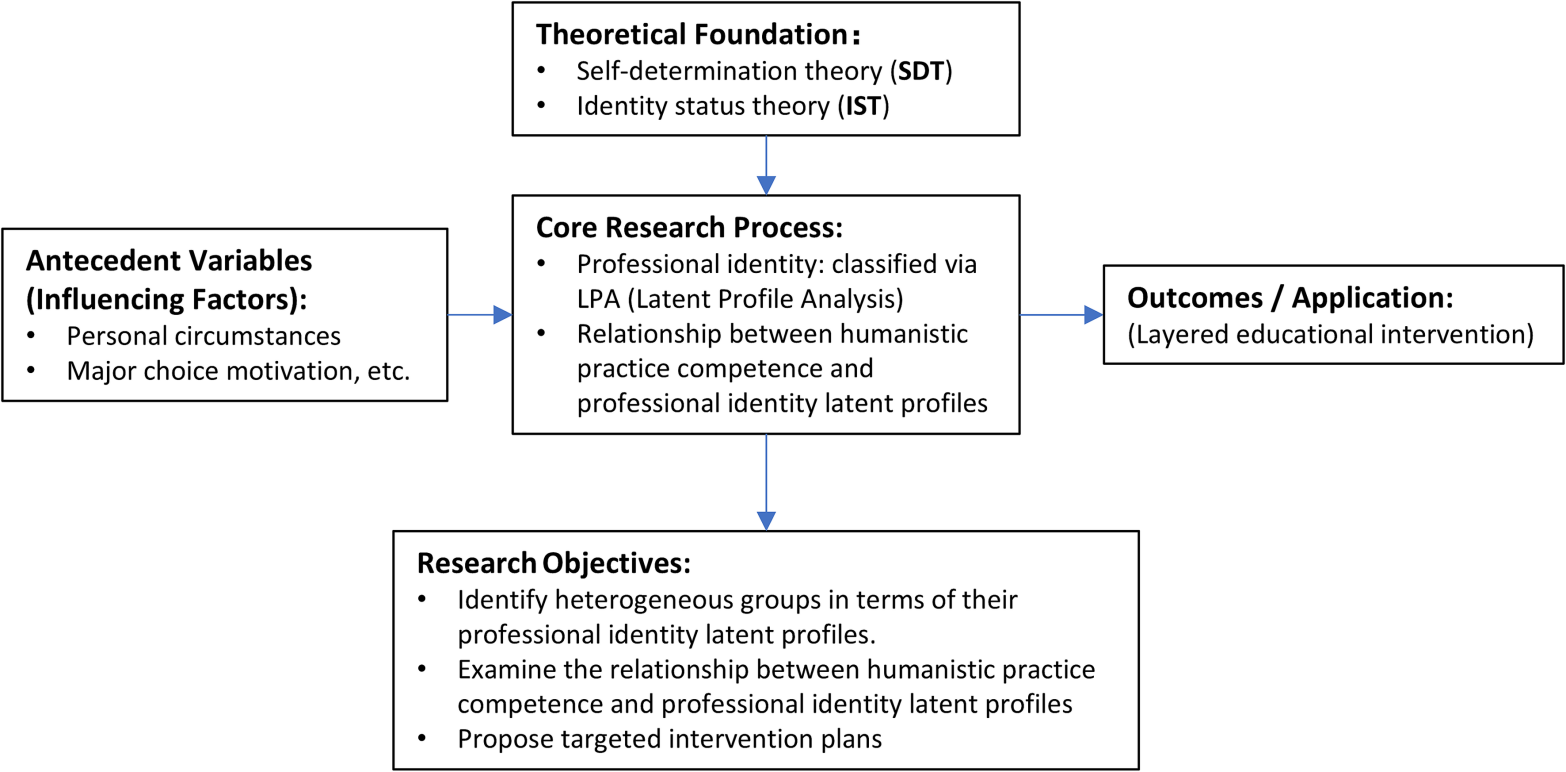
The research framework of this study.

Based on this, we propose the research framework of this study (as illustrated in the Figure 1). Grounded in SDT and IST, this framework aims to explore the association between the professional identity of nursing undergraduates (categorized into distinct latent profiles through Latent Profile Analysis) and their humanistic practice competency. We hypothesize that nursing interns classified into different latent profiles—shaped by varying motivations, competencies, and stages of identity development—will exhibit systematic differences in their humanistic practice competency. This study will first identify these heterogeneous profiles, then compare their competency differences and analyze key influencing factors, with the ultimate goal of providing empirical evidence for developing layered and precise educational intervention strategies.

## 2 Materials and methods

### 2.1 Participants

Using convenience sampling, undergraduate nursing students from the Shanghai University of Medicine & Health Sciences interning who were completing clinical rotations across various departments at tertiary hospitals in Shanghai between December 2023 and April 2025 were recruited. Inclusion criteria were as follows: (1) full-time undergraduate students majoring in nursing at the university;(2) undergoing formal clinical internship at tertiary hospitals in Shanghai with an internship duration of ≥1 month; (3) having completed all basic nursing courses and professional core courses (e.g., Medical Nursing, Surgical Nursing) with no failed courses; (4) being able to independently read and understand the questionnaire content, and complete the survey voluntarily; (5) providing written informed consent after being fully informed of the research purpose, procedures, and rights. Exclusion criteria were as follows: (1)being on continuous leave or absent from internship for more than 1 month during the clinical internship(affecting the validity of clinical experience data);(2)having a history of severe mental illness, cognitive impairment, or communication disorders that affect questionnaire completion; (3) withdrawing from the study midway or refusing to cooperate with questionnaire collection (e.g., incomplete filling, perfunctory responses); (4) violating research ethics or failing to abide by the informed consent agreement. The study included a total of 16 independent variables. The sample size was determined to be 10–20 times the number of independent variables (11). Considering a 20% invalid questionnaire rate, the estimated required sample size was 192–384 cases. All nursing interns signed informed consent forms and voluntarily participated in the study.

### 2.2 Survey instruments

#### 2.2.1 General information questionnaire

The general information questionnaire was self-developed by the researchers with reference to the relevant literature, mainly including participants’ gender, family residence, only-child status, university leadership role, university-level organization membership, grade point average, family economic status, medical staff in family, participation in profession medical volunteer activities, participation in profession medical social practice, reasons for choosing nursing as a major, first choice for a major, current appreciation for the nursing profession, and participation in nursing skill competitions.

#### 2.2.2 Nurse humanistic practice competence assessment scale (NHPCAS)

Developed by Yan (12), this scale comprises 26 items across five dimensions—self-management ability (3 items), humanistic care practice ability (10 items), ethical and legal practice ability (3 items), interpersonal communication ability (6 items), and psychological adjustment ability (4 items)—all with positive descriptions. The items are rated on a 5-point Likert scale (1 = “totally inconsistent” to 5 = “totally consistent”), with a total score range of 26–130. Psychometric validation from the original study (12) demonstrated strong content validity (S-CVI = 0.908), construct validity (KMO = 0.887; CFA confirmed the five-factor structure with good fit: χ^2^/df = 2.667, RMSEA = 0.074), and criterion validity (correlation with Caring Ability Inventory: r = 0.936, *p* < 0.001). Reliability was high (Cronbach’s *α* = 0.913; split-half = 0.841). In this study, the scale showed excellent internal consistency (α = 0.968).

#### 2.2.3 Nurse professional identity assessment scale (NPIAS)

Developed by Liu et al. (13), this scale contains 30 items across five dimensions: professional cognitive evaluation (9 items), professional social skills (6 items), professional social support (6 items), professional frustration coping (6 items), and professional self-reflection (3 items). All items are positively described and rated on a 5-point Likert scale (1 = “totally inconsistent” to 5 = “totally consistent”), with the total score ranging from 30 to 150. Professional identity is classified as follows: high (121–150 points), moderate (91–120 points), low–moderate (61–90 points), and low (30–60 points). The original scale (13) exhibited strong construct validity, with EFA extracting five factors explaining 58.75% of the variance and CFA indicating good model fit (χ^2^/df = 1.85). Internal consistency was excellent (*α* = 0.938). In this study, the scale maintained high reliability (α = 0.967).

### 2.3 Data collection and processing

An online questionnaire was distributed via the Wenjuanxing survey platform. To ensure no missing answers, all questions were set as compulsory questions. Each IP address was restricted to one response to maintain data quality. A standardized instruction was included to explain the research purpose and relevant concepts. After systematic training in questionnaire survey methods, the researchers distributed the questionnaire via WeChat groups for nursing students. Data were directly downloaded from the Wenjuanxing backend, and each questionnaire was manually checked to exclude invalid or illogical responses. Since all questions were set as mandatory, participants could only submit the questionnaire after completing all items, so there were no partially completed questionnaires with partial missing data in this study. Missing data were solely caused by invalid responses (e.g., illogical answers), which were excluded through manual screening. A total of 317 questionnaires were distributed and 295 valid questionnaires were recovered after excluding invalid data, yielding a valid response rate of 93.06%. It should be acknowledged that the online distribution method (via WeChat) and the convenience sampling approach may introduce potential selection bias. For instance, students more active on social media or those who hold stronger opinions on the research topic might have been more likely to participate. To mitigate this bias, the research team ensured that the survey invitation was disseminated across all relevant internship WeChat groups to cover as wide a pool of potential respondents as possible. Furthermore, to ensure response quality, questionnaires with completion times beyond reasonable limits were excluded. Based on a pre-test (n = 20), the average completion time was 12 min. Therefore, responses completed in less than 2 min (indicating careless responding) or more than 30 min (suggesting significant interruption) were discarded to enhance data authenticity.

### 2.4 Statistical analysis

Mplus 8.3 software was used for LPA of Nurse Professional Identity Assessment Scale scores, starting with a single-class model and gradually increasing the number of classes to identify the best-fitting model through comprehensive comparison. Model fit indices included: (1) the Akaike information criterion, Bayesian information criterion, and adjusted Bayesian information criterion, with smaller values indicating better fit; (2) entropy, representing classification accuracy (range: 0–1, with higher values indicating better accuracy; ≥ 0.7 is generally considered excellent); and (3) Lo–Mendell–Rubin likelihood ratio test and bootstrapped likelihood ratio test, where a significant *p* < 0.05 indicated that the k-class model was significantly better than the (k-1)-class model. The final model selection followed this comprehensive decision-making process: First, priority was given to statistical indices, including the decreasing trend of information criteria, high entropy value, and significant *p*-values of the likelihood ratio tests; Second, it was ensured that the sample size proportion of each latent class was reasonable (typically recommended to be >5%); Finally, theoretical knowledge and practical significance were combined to evaluate whether the characteristics of each class were clear and interpretable. SPSS 21.0 software (IBM Corp., Armonk, NY, United States) was used for data processing, including one-way analysis of variance and the chi-square and Kruskal–Wallis H tests for univariate analysis, as well as logistic regression analysis to identify influencing factors of latent profiles of nursing professional identity. The significance level was set at *α* = 0.05.

### 2.5 Ethical considerations

Before starting the survey, we explained to the students the purpose of the study and time required and assured them that the information obtained would be used only for data analysis. The students were informed of their right to withdraw from the study at any time, and that the survey could only be started after obtaining their informed consent. The study was approved by the ethical approval board of School of Nursing and Health Management, Shanghai University of Medicine & Health Sciences (ethical approval number: 2024-bkskt-02-310108200203012825).

## 3 Results

### 3.1 Demographic and background characteristics of participants

The age of the 295 undergraduate nursing interns was (18–27) years (21.89 ± 1.06), with other general characteristics shown in Table 1.

**Table 1:** General characteristics of undergraduate nursing interns and univariate analysis of latent profiles of the nurse professional identity assessment scale (*n* = 295).

Variables	Total (*N* = 295)	Low-level group (*n* = 104)	Moderate-level group (*n* = 146)	High-level group (*n* = 45)	Statistic	*p*-value
Gender					2.756 ^(1)^	0.252
Men	46 (15.60)	21 (20.19)	20 (13.70)	5 (11.11)		
Women	249 (84.40)	83 (79.81)	126 (86.30)	40 (88.89)		
Family residence					0.428 ^(1)^	0.807
Rural	207 (70.17)	71 (68.27)	105 (71.92)	31 (68.89)		
Urban	88 (29.83)	33 (31.73)	41 (28.08)	14 (31.11)		
Only child					4.215 ^(1)^	0.122
Yes	181 (61.36)	70 (67.31)	81 (55.48)	30 (66.67)		
No	114 (38.64)	34 (32.69)	65 (44.52)	15 (33.33)		
University leadership role					0.07 ^(1)^	0.966
Yes	95 (32.20)	34 (32.69)	46 (31.51)	15 (33.33)		
No	200 (67.80)	70 (67.31)	100 (68.49)	30 (66.67)		
University-level organization membership					7.81 ^(1)^	0.020
Yes	122 (41.36)	32 (30.77)	67 (45.89)	23 (51.11)		
No	173 (58.64)	72 (69.23)	69 (54.11)	22 (48.89)		
Grade point average					1.044 ^(2)^	0.593
3.7–4.0	79 (26.78)	29 (27.88)	38 (26.02)	12 (26.67)		
3.3–3.7	129 (43.73)	39 (37.50)	66 (45.21)	24 (53.33)		
2.0–3.3	87 (29.49)	36 (34.62)	42 (28.77)	9 (20.00)		
Family economic status					0.488 ^(2)^	0.783
Good	129 (43.73)	44 (42.31)	66 (45.21)	19 (42.22)		
Average	151 (51.19)	55 (52.88)	74 (50.68)	22 (48.89)		
Poor	15 (5.08)	5 (4.81)	6 (4.11)	4 (8.89)		
Medical staff in family					0.048 ^(1)^	0.976
Yes	80 (27.12)	29 (27.88)	39 (26.71)	12 (26.67)		
No	215 (72.88)	75 (72.12)	107 (73.29)	33 (73.33)		
Participation in professional medical volunteer activities					9.567 ^(1)^	0.008
Yes	155 (52.54)	42 (40.38)	87 (59.59)	26 (57.78)		
No	140 (47.46)	62 (59.62)	59 (40.41)	19 (42.22)		
Participation in professional medical social practice					2.808 ^(1)^	0.246
Yes	61 (20.68)	16 (15.38)	35 (23.97)	10 (22.22)		
No	234 (79.32)	88 (84.62)	111 (76.03)	35 (77.78)		
Reasons for choosing nursing as a major					20.538 ^(1)^	0.002
Interest in nursing	59 (20.00)	17 (16.35)	24 (16.43)	18 (40.00)		
Easy employment	137 (46.44)	48 (46.15)	72 (49.32)	17 (37.78)		
Parental advice	70 (23.73)	22 (21.15)	40 (27.40)	8 (17.78)		
Major transfer	29(9.83)	17 (16.35)	10 (6.85)	2 (4.44)		
First choice for a major					9.643 ^(1)^	0.008
Yes	190 (64.41)	58 (55.77)	95 (65.07)	37 (82.22)		
No	105 (35.59)	46 (44.23)	51 (34.93)	8 (17.78)		
Current appreciation for the nursing profession					45.679 ^(1)^	< 0.001
Yes	159 (53.90)	30 (28.85)	92 (63.01)	37 (82.22)		
No	136 (46.10)	74 (71.15)	54 (36.99)	8 (17.78)		
Participation in nursing skill competitions					5.098 ^(1)^	0.078
No	256 (86.78)	95 (91.35)	126 (86.30)	35 (77.78)		
Yes	39 (13.22)	9 (8.65)	20 (13.70)	10 (22.22)		

^(1)^χ2 value; ^(2)^Kruskal-Wallis H value.

### 3.2 Score status of the NPIAS and NHPCAS for undergraduate nursing interns

The participants’ average score on the NPIAS was (111.33 ± 18.05) points, the average score on the NHPCAS was (101.49 ± 14.94) points, and the score status of each dimension is shown in Table 2.

**Table 2:** Score status of the NPIAS and NHPCAS for undergraduate nursing interns (points, Mean ± SD).

Variables	Total (*N* = 295)	Low-level group (*n* = 104)	Moderate-level group (*n* = 146)	High-level group (*n* = 45)	F-value	*p*-value
Professional cognitive evaluation	32.15 ± 6.49	26.03 ± 4.41	33.53 ± 3.33	41.82 ± 3.00	305.351	< 0.001
Professional social support	23.01 ± 3.53	19.63 ± 2.16	23.72 ± 1.75	28.53 ± 1.55	373.393	< 0.001
Professional social skills	21.85 ± 4.10	18.02 ± 2.35	22.58 ± 2.25	28.38 ± 1.67	360.899	< 0.001
Professional frustration coping	22.97 ± 3.62	19.31 ± 2.01	23.84 ± 1.60	28.64 ± 1.55	483.100	< 0.001
Professional self-reflection	11.34 ± 1.89	9.61 ± 1.23	11.69 ± 1.02	14.2 ± 0.94	293.935	< 0.001
Total score of NPIAS	111.33 ± 18.05	92.6 ± 7.87	115.36 ± 6.96	141.58 ± 7.33	741.492	< 0.001
Self-management ability	11.14 ± 1.96	9.14 ± 1.12	11.05 ± 1.33	13.48 ± 1.52	171.937	<0.001
Humanistic care practice ability	39.61 ± 5.90	31.32 ± 2.57	39.98 ± 2.61	47.3 ± 2.49	624.197	<0.001
Ethical and legal practice ability	11.87 ± 1.94	9.32 ± 1.15	11.98 ± 1.00	14.29 ± 0.97	375.130	<0.001
Interpersonal communication ability	23.3 ± 3.74	18.38 ± 1.77	23.37 ± 1.88	28.29 ± 1.63	486.965	<0.001
Psychological adjustment ability	15.57 ± 2.44	12.39 ± 1.15	15.56 ± 1.13	18.92 ± 1.17	525.806	<0.001
Total score of NHPCAS	101.49 ± 14.94	80.55 ± 5.22	101.94 ± 5.69	122.27 ± 6.13	869.570	<0.001

### 3.3 Results of the LPA of the NPIAS

**Figure 2:**
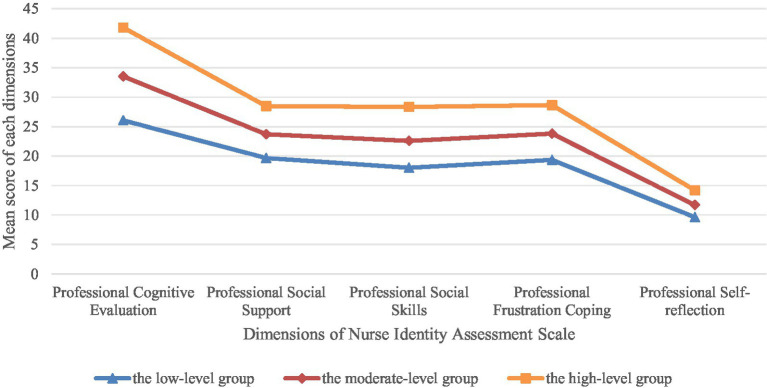
Latent profiles of the NPIAS for nursing interns. Line plots of five professional identity dimension scores across three latent profiles (low/moderate/high).

Three models were fitted in this study, with fit indices shown in Table 3. Starting from a single-class model, the values of the Akaike information criterion, Bayesian information criterion, and adjusted Bayesian information criterion gradually decreased as the number of classes increased. When the number of classes was three, entropy was high, and both Lo–Mendell–Rubin likelihood ratio test and bootstrapped likelihood ratio test values were statistically significant (*p* < 0.001). The model comparison process was as follows: Compared to the single-class model, all information criteria (AIC, BIC, aBIC) for the two-class model decreased significantly, the entropy value reached 0.882, and both the LMRT and BLRT were significant (*p* < 0.001), indicating that the two-class model was superior to the single-class model. Furthermore, the information criteria for the three-class model decreased further, the entropy value increased to 0.918, indicating excellent classification accuracy, and the LMRT and BLRT were again significant (*p* < 0.001), demonstrating that the three-class model represented a significant improvement over the two-class model. When attempting a four-class model, although the information criteria continued to decrease slightly and the LMRT and BLRT remained significant, the entropy value decreased slightly (0.910), and the sample proportion of one class was only 12.90%, approaching the lower acceptable limit. Its theoretical distinction and practical guidance significance were less clear and definitive than those of the three-class solution. Therefore, based on the following core justifications, we ultimately determined the three-class model as the optimal solution. (1) Excellent statistical indices: the three-class model had lower information criteria, excellent entropy (0.918), and significant likelihood ratio test results, indicating good statistical fit; (2) Reasonable class sizes: the sample proportions of all classes were greater than 5%, with the smallest class accounting for 15.25%, ensuring the stability and representativeness of the classes; (3) Clear theoretical meaning: the three classes (Low, Moderate, High) corresponded well to different stages of professional identity development, possessing distinct characteristics and clear implications for practical intervention. Based on scores on the NPIAS, the classes were named as follows: Class I students had significantly low scores on all dimensions and were named the low-level group, with 104 cases (35.25%); Class II students had moderate scores on all dimensions and were named the moderate-level group, with 146 cases (49.50%); and Class III students had high scores on all dimensions and were named the high-level group, with 45 cases (15.25%), as shown in Figure 2 and Table 2.

**Table 3:** Fit indices of different latent profiles.

Number of classes	AIC	BIC	aBIC	Entropy	*p*-value (LMRT)	*p*-value (BLRT)	Class proportion (%)
1	8014.054	8050.924	8019.211	-—	—	—	—
2	7255.174	7314.165	7263.424	0.882	< 0.001	< 0.001	0.531/0.469
3	6774.753	6855.867	6786.098	0.918	< 0.001	< 0.001	0.3525/0.495/0.1525
4	6601.533	6704.769	6615.972	0.91	0.002	< 0.001	0.206/0.310/0.355/0.129

AIC, Akaike information criterion; BIC, Bayesian information criterion; aBIC, adjusted Bayesian information criterion; LMRT, Lo–Mendell–Rubin likelihood ratio test; BLRT, Bootstrapped likelihood ratio test.

### 3.4 Univariate analysis of latent profiles of the NPIAS

Univariate analysis showed significant differences in latent categories of professional identity among undergraduate nursing interns across demographic and background variables (Table 1). This indicates that professional identity level is closely related to students’ professional choice motivation, practical participation, and organizational experience, providing important clues for subsequent multivariate analysis.

### 3.5 Multivariate analysis of the latent profiles of the NPIAS

A logistic regression model was constructed using the latent profiles of the NPIAS as the dependent variable (with “low” as the reference group). In SPSS operations, categorical variables were automatically set as dummy variables. Variable coding was as follows: (1) university-level organizational membership: yes = 1, no = 2; (2) participation in professional medical volunteer activities: yes = 1, no = 2; (3) first choice for a major: yes = 1, no = 2; (4) current appreciation for the nursing profession: yes = 1, no = 2; (5) reasons for choosing nursing as a major: interest in nursing (Z1 = 0, Z2 = 0, Z3 = 0, Z4 = 0), easy employment (Z1 = 0, Z2 = 1, Z3 = 0, Z4 = 0), parental advice (Z1 = 0, Z2 = 0, Z3 = 1, Z4 = 0), and major transfer (Z1 = 0, Z2 = 0, Z3 = 0, Z4 = 1). Dimensional scores of the Nurse Professional Identity Assessment Scale were entered as continuous variables. The results showed that compared with the low-level group, students who chose the major owing to “current appreciation for the nursing profession,” “parental advice,” and had strong self-management ability were more likely to belong to the moderate-level group (odds ratio [OR] = 4.294; OR = 5.695; OR = 2.072, respectively). Students who “current appreciation for the nursing profession” and who possessed strong self-management ability and ethical and legal practice abilities were more likely to belong to the high-level group (OR = 12.688; OR = 5.047; OR = 3.141, respectively), as shown in Table 4.

**Table 4:** Logistic regression analysis of latent profiles of the NPIAS among undergraduate nursing interns.

Variables	Moderate-level group					
	Regression coefficient	SE	Wald	OR	95% CI	*p*-value
Constant	−13.941	2.286	37.198	—	—	< 0.001
University-level organizational membership	0.245	0.549	0.2	1.278	0.436–3.745	0.655
Participation in professional medical volunteer activities	0.457	0.527	0.753	1.579	0.562–4.434	0.386
First-choice major	−0.022	0.519	0.002	0.978	0.354–2.704	0.966
Current appreciation for the nursing profession	1.457	0.518	7.925	4.294	1.557–11.842	0.005
Reference: Interest in nursing						
Easy employment	0.498	0.657	0.576	1.646	0.454–5.963	0.448
Parental advice	1.74	0.773	5.066	5.695	1.252–25.905	0.024
Major transfer	0.17	0.896	0.036	1.185	0.205–6.858	0.849
Self-management ability	0.728	0.214	11.547	2.072	1.361–3.154	0.001
Humanistic care practice ability	−0.082	0.096	0.723	0.922	0.764–1.112	0.395
Ethical and legal practice ability	0.428	0.241	3.143	1.533	0.956–2.46	0.076
Interpersonal communication ability	−0.086	0.139	0.383	0.918	0.699–1.205	0.536
Psychological adjustment ability	0.356	0.207	2.953	1.428	0.951–2.142	0.086
Variables	High-level group					
	Regression coefficient	SE	Wald	OR	95% CI	*p*-value
Constant	−48.682	7.787	39.087	—	—	< 0.001
University-level organizational membership	0.014	1.085	< 0.001	1.014	0.121–8.503	0.989
Participation in professional medical volunteer activities	−1.415	1.216	1.355	0.243	0.022–2.632	0.244
First-choice major	2.743	1.415	3.757	15.532	0.97–248.776	0.053
Current appreciation for the nursing profession	2.541	1.033	6.043	12.688	1.674–96.182	0.014
Reference: Interest in nursing						
Easy employment	0.929	1.141	0.663	2.531	0.271–23.671	0.416
Parental advice	1.747	1.383	1.596	5.738	0.382–86.271	0.206
Major transfer	0.454	2.592	0.031	1.575	0.01–253.218	0.861
Self-management ability	1.619	0.402	16.224	5.047	2.296–11.094	< 0.001
Humanistic care practice ability	0.059	0.228	0.066	1.061	0.678–1.659	0.797
Ethical and legal practice ability	1.144	0.489	5.484	3.141	1.205–8.184	0.019
Interpersonal communication ability	−0.086	0.345	0.061	0.918	0.467–1.806	0.804
Psychological adjustment ability	0.614	0.38	2.606	1.847	0.877–3.892	0.106

The low-level group was used as the reference for the dependent variable. SE, standard error; CI, confidence interval.

## 4 Discussion

### 4.1 Current status and latent profile characteristics of professional identity among undergraduate nursing interns

Participants’ total score on the NPIAS was (111.33 ± 18.05), falling within the moderate level (91–120) points. LPA identified three latent categories: low-level group (35.25%), moderate-level group (49.50%), and high-level group (15.25%). This distribution indicates significant heterogeneity in the professional identity of undergraduate nursing interns, predominantly at moderate to low levels. This pattern is consistent with domestic findings (14), yet it also finds resonance in international contexts. This finding parallels the well-documented high global prevalence of nurse burnout in Western healthcare systems (15). However, the similar outcomes appear to stem from distinct underlying cultural drivers.

Building on our cross-sectional findings, we propose a conceptual pathway to illustrate how professional identity evolves. We propose a conceptual pathway, framed by IST (3), to illustrate how professional identity evolves. The pathway begins with external influences (e.g., parental advice, major transfer), which may create a cognitive—affective misalignment—a disconnect between students’ actions and personal values. This mismatch can lead to emotional resistance and stagnant development (the low group), a state analogous to Identity Diffusion in psychological theory. For others, positive environmental interactions (e.g., volunteer activities, clinical successes) can trigger a transitional phase of exploration, reminiscent of Moratorium. In this phase, which characterizes the moderate group, professional identity is fragile and remains dependent on external factors. The endpoint is internalization, where professional values are fully integrated into the self-concept. This integration creates a self-sustaining cycle that enhances motivation and competence, characterizing the high group and corresponding to the Identity Achievement status. This progression from external regulation to integrated motivation is also supported by the continuum of motivation described in SDT (2).

Students in the low-level group (35.25%) demonstrated significantly lower scores across all dimensions. For instance, their professional cognitive evaluation score was only (26.03 ± 4.41). This group was characterized by a high prevalence of passive entry into the profession (16.35%) admitted via “major transfer” and (71.15%) lacking interest in the nursing profession, *p* < 0.001. It forms a negative cycle of “passive choice → emotional resistance → cognitive blank” (16). This phenomenon of disengagement and low motivation finds a parallel in the ‘burnout’ and ‘professional disillusionment’ described among nursing interns in Western contexts (17). However, while the outcomes are phenomenologically similar, their etiological drivers are culturally distinct. In our Chinese cohort, the genesis often lies in extrinsic, pre-professional factors such as familial influence (‘parental advice’) or institutional mechanisms (‘major transfer’), representing a form of ‘passive entry’. Conversely, Western literature frequently attributes similar low identification to factors intrinsic to the clinical environment itself, such as overwhelming workload pressure and exposure to negative workplace dynamics (18). This crucial distinction highlights that our findings are not merely a replication of a global issue but reveal a culturally specific pathway to professional identity crisis, rooted in initial selection motives rather than subsequent clinical experiences. The low-level group illustrates a potential breakdown in the identity formation pathway, where a lack of initial intrinsic motivation hinders the development of a professional identity necessary for sustained clinical engagement. Interventions should focus on emotional support. Narrative pedagogy is specifically suited here, as stories can bypass cognitive resistance and activate emotional identification; the power of narrative to foster empathy and connection is well-documented [e.g., (19)]. This is paired with micro-goal mentoring to provide immediate accomplishments that build self-efficacy, a key driver of behavioral change according to Social Cognitive Theory (7). Together, these strategies form a reinforcing circle essential for initiating change. The primary theoretical aim here is to address the fundamental ‘autonomy’ deficit noted in SDT by creating a safe space for emotional exploration (via narrative) and to build ‘competence’ through achievable successes (via micro-goals), thereby planting the seed for intrinsic motivation in a group characterized by its initial absence.

Students in the moderate-level group (49.50%) performed better on dimensions such as professional frustration coping (23.84 ± 1.60) and professional social skills (22.58 ± 2.25) but had weaker professional self-reflection ability (11.69 ± 1.02). Among this group, 59.59% had participated in professional volunteer activities, and 27.40% chose the major owing to “parental advice.” The moderate-level group represents a crucial transitional phase. Their identity is significantly influenced by external environments and lacks long-term planning. In the framework of IST, this group closely aligns with the ‘Moratorium’ status, actively exploring but not yet having made a firm commitment. In the proposed pathway, this group’s stability is precarious; without intervention to foster internalization, they may not progress to a high-level, self-sustaining identity. To facilitate this internalization process, we recommend interventions centered on reflective structuring. The key objective is to convert positive external experiences into stable internal beliefs. Structured reflective practice is critical here. Guided reflection is an established method for enhancing self-awareness and bridging theory with practice in healthcare education [e.g., (1)]. Furthermore, facilitated narrative sharing helps students construct a coherent “personal-professional” narrative, thereby transforming a passive, externally-driven identity into an active, intrinsic one, a process that aligns with the concept of narrative identity construction (20). The overarching goal of these interventions, from an SDT perspective, is to facilitate the internalization process—transforming externally regulated motivations into more integrated forms. By providing structure for reflection (enhancing ‘competence’) and ownership over one’s narrative (fostering ‘autonomy’), we aim to help students move from a fragile, externally-dependent identity toward ‘Identity Achievement’.

Students in the high-level group (15.25%) had significantly higher scores on dimensions such as professional cognitive evaluation (41.82 ± 3.00) and professional social support (28.53 ± 1.55), which reflected their deep understanding of nursing values, active seeking of team support, and flexible coping with professional challenges. Further analysis showed that 82.22% of this group “currently appreciated the nursing profession,” and 40.00% chose the major owing to “interest in nursing,” significantly higher than the low group (28.85 and 16.35%, respectively). This suggests that intrinsic motivation (interest-driven) is a core driver of high professional identity (2). For this group, dual mentorship extends their career vision, linking their role to broader paths and thus reinforcing long-term commitment, a benefit noted in mentorship literature (16). “Our recommendation of a ‘dual mentorship’ system for high-achieving students is supported by successful implementations in countries with advanced nursing education systems. For example, the Clinical Scholar Model in the United States has demonstrated that pairing students with both a clinical and an academic mentor significantly enhances research leadership and career planning capabilities (21), aligning perfectly with our goal of sustaining development for the high-level group.” Theoretically, this strategy directly supports the SDT need for ‘autonomy’ by expanding students’ sense of choice and future pathways, and ‘relatedness’ by deepening their connection to the professional community. Furthermore, engaging them in scientific research innovation allows them to deepen their professional interest by transforming clinical problems into scholarly inquiry, which powerfully satisfies the SDT need for ‘competence’ (mastery) and further reinforces their ‘autonomous’ engagement with the profession (2).

### 4.2 Analysis of influencing factors of the nurse professional identity

#### 4.2.1 SDT in identity formation: from external advice to internal identification

Students who chose nursing as a major owing to “parental advice” were more likely to belong to the moderate-level group, while those who “Current appreciation for the nursing profession” were more likely to belong to the high-level group. This aligns with self-determination theory (2): intrinsic motivation (interest) fosters deep identification by satisfying autonomy needs. Students with strong interest are more willing to actively overcome professional challenges, such as improving skills through self-study or seeking mentor support, while external motivation (parental advice) may lead to fragile identity. Colleges should implement pre-admitting interventions such as nursing career experience camps and open days to help prospective students clarify career goals and convert external expectations into internal interest. Innovative modules such as narrative nursing can also be integrated into core curricula. By sharing real patient stories and nursing experiences, educators can demonstrate the social value and emotional rewards of nursing, thereby enhancing professional attractiveness and strengthening intrinsic motivation among the moderate-level group students (22). For students influenced by parental advice, individualized motivation counseling is recommended to help them explore personal interests, align external expectations with self-development, and foster a shift from passive acceptance to active identification.

#### 4.2.2 Self-management and ethical practice as pillars of professional identity

Higher scores on the self-management and ethical practice ability dimensions of humanistic practice competence increased the likelihood of belonging to the high-level group. This finding suggests that these competencies are not merely supplementary but may be fundamental pillars supporting the development of a robust professional identity.

Theoretically, this relationship can be understood through the lens of Self-Determination Theory (2). Effective self-management directly fulfills the need for autonomy by fostering a sense of personal control over one’s learning and clinical responsibilities, which in turn helps mitigate burnout—a known barrier to identity formation (23). Conversely, a firm grounding in ethical practice sustains the need for relatedness by solidifying the nurse’s commitment to the core values of the profession, such as patient advocacy and privacy protection, thereby strengthening the sense of belonging to the nursing community and reinforcing professional responsibility (24).

Consequently, nursing education should prioritize the systematic cultivation of these competencies. We recommend building a comprehensive evaluation system that positions self-management and ethical practice as core indicators of humanistic competence. To achieve this, nursing curricula should integrate structured self-management training, including time management, stress regulation, and goal setting. These components should be reinforced through clinical simulations. Simultaneously, ethical reasoning must be strengthened through the analysis of realistic dilemmas using standardized patients, an approach shown to effectively bridge theory and practice in healthcare education [e.g., (25)], ensuring that professional responsibility is consistently reinforced throughout clinical internship.

## 5 Limitations

This study has several limitations that also outline clear directions for future research. First, the generalizability of the findings is constrained by the convenience sampling strategy and the recruitment of participants from a single university in Shanghai. Consequently, the applicability of our findings to nursing intern populations in other regions of China or with different socio-cultural and educational backgrounds may be limited. Future studies should therefore expand the sample size and diversity to validate these results across different geographical and educational contexts. Second, the cross-sectional design precludes the examination of causal relationships and dynamic development. A longitudinal approach, tracking interns at multiple timepoints (e.g., pre-, mid-, and post-internship), is needed to elucidate the trajectory and causal mechanisms of professional identity formation. Third, the reliance on self-reported data introduces the risk of social desirability and self-assessment biases. To overcome this, subsequent research would benefit from employing mixed methods, incorporating objective evaluations (e.g., preceptor ratings, clinical performance data) and in-depth qualitative interviews to triangulate findings and enhance validity. Finally, our model did not incorporate several important contextual variables. Specifically, the role of specific Chinese cultural constructs (e.g., collectivism, occupational hierarchy) and key environmental factors (e.g., clinical teaching styles, hospital culture) were not examined. Future work should explicitly incorporate these factors as predictive or moderating variables to unravel their impact on professional identity within the broader socio-cultural and organizational context.

## 6 Conclusion

Latent Profile Analysis (LPA) provided a unique and powerful lens for this study, moving beyond variable-centered approaches to reveal three distinct, actionable profiles of professional identity among nursing interns: the low-level, moderate-level, and high-level groups. This person-centered classification demonstrates that professional identity is not a uniform trait but a heterogeneous construct, fundamentally shaped by a combination of motivational sources (intrinsic vs. external) and core competencies (self-management and ethical practice). The clear delineation of these profiles offers a direct and compelling mandate for nursing education. To translate these findings into practice, educators must integrate stratified intervention strategies directly into the nursing curriculum. This includes embedding narrative pedagogy and micro-goal mentoring for the low-level group, structured reflective practice for the moderate-level group, and research innovation opportunities for the high-level group. Ultimately, adopting this profile-based, personalized approach is crucial for moving beyond a one-size-fits-all model and effectively fostering a robust professional identity across the entire student population.

## Data Availability

The raw data supporting the conclusions of this article will be made available by the authors, without undue reservation.
